# LONGITUDINAL ASSOCIATIONS BETWEEN PERCEIVED STRESS AND MEMORY COMPLAINTS

**DOI:** 10.1093/geroni/igz038.807

**Published:** 2019-11-08

**Authors:** Tyler Bell, Tyler Bell, Jacqueline Mogle, Nikki Hill

**Affiliations:** 1 Penn State College of Nursing, University Park, Pennsylvania, United States; 2 Pennsylvania State University, University Park, Pennsylvania, United States

## Abstract

Memory complaints increase cognitive decline but show weak concurrent associations with objective memory. Instead, affect might underlie some memory complaints and their impact on future cognition. Perceived stress influences cognitive performance, but temporal associations with memory complaints is unknown. We therefore explored longitudinal relationships between perceived stress and memory complaints among cognitively normal older adults. From the Einstein Aging Study (n=507, Mage=77.88, 63.30% female; 73.21% White), multilevel models examined bi-directional concurrent and one-year-lagged associations between within-person changes in perceived stress and memory complaints (frequency of forgetting, perceived one-year memory decline, perceived ten-year memory decline). Perceived stress positively covaried with memory complaints. Looking at lagged effects, only frequency of forgetting predicted next-year perceived stress. Higher frequency of forgetting thus increases perceived stress while perceived decline associates with current perceived stress. Reframing perceptions of forgetfulness might reduce stress in cognitively intact older adults, which in turn may benefit cognition long term.

